# Prevalence, trend, and inequality of prolonged exclusive breastfeeding among children aged 6–23 months old in India from 1992–2021: A cross-sectional study of nationally representative, individual-level data

**DOI:** 10.7189/jogh.14.04026

**Published:** 2024-02-09

**Authors:** Zekun Chen, Smriti Sharma, Shaoru Chen, Rockli Kim, S V Subramanian, Zhihui Li

**Affiliations:** 1 Vanke School of Public Health, Tsinghua University, Beijing, China; 2 Mother Infant and Young Child Nutrition, Tata Trusts, Delhi, India; 3 Division of Health Policy and Management, College of Health Science, Korea University, Seoul, South Korea; 4 Interdisciplinary Program in Precision Public Health, Department of Public Health Sciences, Graduate School of Korea University, Seoul, South Korea; 5 Department of Social and Behavioral Sciences, Harvard T.H. Chan School of Public Health, Boston, Massachusetts, USA; 6 Harvard Center for Population and Development Studies, Cambridge, Massachusetts, USA; 7 Institute for Healthy China, Tsinghua University, Beijing, China

## Abstract

**Background:**

Prolonged exclusive breastfeeding (PEB) for children older than six months old is a threat to appropriate complementary feeding practices. This study aims to examine the trend of PEB among children aged 6–23 months in India.

**Methods:**

We adopted five waves of National Family Health Survey (NFHS) data between 1992–93 and 2019–21. PEB was defined as children aged six months and above currently consuming breastmilk as the only source of energy, protein and micronutrients. We generated descriptive statistics and a series of multivariable logistic regressions to estimate the prevalence and trend in the PEB rate. Moreover, we assessed how child age and socioeconomic factors (i.e. child gender and age, place of residence, household wealth, and maternal education) were related with PEB using mutually and single-adjusted model.

**Results:**

There were 184 891 Indian children aged 6–23 months old included in this study with 48.0% being female. We found that the proportion of PEB increased from 4.3% in 1992 to 7.7% in 2021, of which the rate for children aged six-eight months rose from 14.0 to 20.1%. Our results showed that children who were from poorer households or with lower-educated mothers were more likely to experience prolonged exclusive breastfed. Take the year of 2019–21 as an example, compared to the households of the richest quintile, children from households of the poorer quintile were significantly more likely to experience PEB, with odds ratio (OR) of 1.33 (95% confidence interval (CI) = 1.09–1.61). Moreover, children with illiterate mothers had 21% higher odds of having prolonged exclusively breastfeeding (OR = 1.21; 95% CI = 1.01–1.44) compared with children with mothers who have college and above education.

**Conclusions:**

PEB among children over six months old is prevalent in India, particularly among children from disadvantaged households. Poverty reduction and maternal education are of great potential importance for policymakers to promote appropriate complementary feeding practice.

Sustainable Development Goal 2 aims to eradicate hunger and ensure universal access to safe, nutritious and sufficient food throughout the year by 2030 [1]. Despite being a significant food producer globally, India bears nearly one-third of the global burden of hunger [2]. Additionally, 80% of infants and young children in India do not meet the minimum dietary diversity, with insufficient consumption of protein-rich foods [3]. To support the growth and development of infants, complementary foods are gradually introduced around the age of six months [4]. The World Health Organization (WHO) provides guidelines for infant and young child feeding practices to guide health care workers and inform policymakers [4–7]. According to these guidelines, infants should be exclusively breastfed for the first six months of life, meaning they should not be fed any other source of milk, water, honey, or other food during this period. Key indicators used to monitor breastfeeding practices include early initiation of breastfeeding, exclusive breastfeeding, and continued breastfeeding. After six months, complementary feeding should be introduced to meet the growing nutritional needs of the child, which cannot be fulfilled by breastmilk alone. It is important to ensure that the timing, safety, adequacy, and appropriateness of feeding is maintained. Commonly used indicators to monitor complementary feeding practices include the introduction of solid, semi-solid or soft foods between six-eight months of age, minimum dietary diversity, minimal meal frequency and minimum adequate diet [7].

As an infant grows, their daily energy, protein, and micronutrient requirements increase. At six-eight months, the daily energy requirement is 600 kilocalorie per day (kcal/d), which increases to 700 kcal/d at 9–11 months and 900 kcal/d at 12–23 months [8]. For a breastfed infant, external foods are necessary to provide a portion of their daily calorific needs. At six-eight months, on-demand breastfed infants require 200 kcal/d from external foods, which represents 33% of their daily calorific needs. At 9–11 months, the requirement increases to 300 kcal/d, representing 43% of their daily calorific needs. Finally, at 12–23 months, the requirement is 550 kcal/d, representing 61% of their daily calorific needs [9]. Indeed, meeting these calorific needs alone does not ensure adequate nutrition. The composition of complementary foods must also be nutritionally adequate, providing a balance of protein, fat, and micronutrients. However, meeting the nutritional needs alongside minimum calorific needs of infants and young children is crucial for their growth and development.

The lack of adequate nutrition among infants and young children can have significant short-term and long-term consequences. The adverse consequences of the late introduction of complementary food in infants' and young children’s growth and development are well documented. Previous studies indicated that untimely supplementation of complementary food during the first six-eight months of life can raise the risk of macro as well as micronutrient deficiencies in infants and young children impacting their growth potential [10,11]. In addition, after six months of age, complementary foods are an important source of iron and other nutrients that are needed for hemoglobin synthesis, accounting for about 95% of iron requirements of children at 12–23 months. If complementary foods are not added in a timely manner, the risk of iron deficiency anemia in infants may increase. Siimes et al. [12] reported that both serum iron and serum ferritin were significantly lower in infants who were exclusively breastfed over six months than those supplemented with complementary foods. The late introduction of complementary foods may even impair immunity as well as brain development, leading to short and long-term consequences further implying the need to focus on food-based measures of child’s nutritional status [13,14]. Prolonged exclusive breastfeeding (PEB) is defined as infants continuing to rely solely on breastmilk beyond the age of six months, which is against recommended infant and young child feeding practices and can deprive the child of the nutrients needed for growth and development. Previous studies have indicated that PEB may increase the risk of atopic dermatitis, food allergies, and anemia in children [15,16].

Delayed introduction of soft, semi-solid, and solid foods to infants at six months of age may be a critical factor contributing to the prevalence of PEB. In India, some mothers may consider breastmilk a substitute for complementary foods, resulting in a higher number of children under PEB [17,18]. Previous studies reported that, in India, a large proportion of women exclusively breastfeed for up to eight months or even longer, especially in rural areas [19]. There is much evidence showing that feeding practice is closely related to the socioeconomic status of the households. Several studies from India and other low- and middle-income countries (LMICs) showed that higher household wealth and better maternal education are significantly associated with a timelier introduction of complementary feeding, better access to nutritious foods, and more diversified foods provided to children [20–23]. Studies also found that the poorer and the less-educated population tended to have less knowledge of child nutrition and proper feeding practices to be performed on infants and young children and, therefore, were less likely to translate the related information into practice [24,25]. However, few studies have assessed trends in socioeconomic inequality in PEB for infants aged 6–23 months in LMICs [26–28], and no such study was conducted in India.

To promote appropriate feeding practices, particularly among the vulnerable population, it is critical to identify which group of children are in the most urgent need of assistance. This study aims to investigate the prevalence of PEB and identify factors that may contribute to its occurrence. To achieve this, five waves of National Family Health Survey (NFHS) data between 1992–93 and 2019–21 were conducted to track the changes in the prevalence of prolonged exclusive breastfeeding among children aged 6–23 months old. Additionally, we will examine the relationship between PEB and socioeconomic factors.

## METHODS

### Data source

Data for this study were obtained from five waves of NFHS conducted in 1992–93 (NFHS-1), 1998–99 (NFHS-2), 2005–06 (NFHS-3), 2015–16 (NFHS-4), and 2019–21 (NFHS-5). Our study followed the Strengthening the Reporting of Observational Studies in Epidemiology (STROBE) reporting guideline. NFHS is a cross-sectional, nationally representative survey that provides nutrition and health information on children, their parents, and households [29]. NFHS used a multistage stratified sampling design. For the rural areas, a two-stage stratified sampling was adopted with the selection of villages followed by the selection of households; for the urban areas, a three-stage sample design was adopted with the selection of cities/towns followed by urban blocks, and finally households [30].

### Study population

The NFHS enrolled a total of 626 087 individuals, with 48 959 from NFHS-1, 33 026 from NFHS-2, 51 555 from NFHS-3, 259 627 from NFHS-4 and 232 920 from NFHS-5, respectively. After removing 38 295 children with no age information and 402 901 children who were not 6–23 months of age, we finally included 184 891 study participants (Figure S1 in the **Online Supplementary Document**)

### Outcome

The NFHS included a 24-hour recall of food and liquid consumed by the children. Solid food includes chicken, duck, other birds, beef, pork, lamb, bread, noodles, other made from grains, potatoes, cassava, or other tubers, pumpkin, carrots, squash, dark green leafy vegetables, eggs, mangoes, papayas, other vitamin fruit, liver, heart, other organs, beans, peas, lentils, nuts, cheese, yogurt, other milk products, oil, fats, butter or other solid or semi-solid food. Liquid food includes all food in liquid form that provides nutrition or energy, such as honey water, sugary water, juice, broth, formula, and other forms of milk other than breastmilk. Water includes water, sugar water, juice, or tea. Exclusive breastfeeding is defined as receiving no food, liquid, or water excluding breastmilk in the last 24 hours according to WHO guidelines [5]. We follow the prior study that defined PEB as the exclusive receipt of breastfeeding among children aged 6–23 months [16]. However, there are also studies that defined PEB including water [15]. To demonstrate the robustness of our findings, we conducted an analysis on children receiving PEB and water in the sensitivity analysis.

### Explanatory variables

We included a series of sociodemographic factors as the explanatory variables. The demographic variables were child age, child gender, and place of residence (rural/urban). Since children of different ages had various demands for complementary feeding, we divided them into four age groups: six-eight months, 9–11 months, and 12–23 months. The child’s gender and place of residence were dichotomous. The socioeconomic variables included household wealth and maternal education, Household wealth was constructed by NFHS based on a selected set of household assets (e.g. consumer items and home attributes) and was in quintiles. Maternal education had four categories, including no education, primary education, secondary education, and higher education.

### Other covariates

A number of covariates were included in this study because they might impact the breastfeeding practices of children. These variables included breastfeeding initiation (≥1 hour (h) of birth /<1 hour of birth), drinking water source (safe/unsafe), sanitation facility (improved/not improved), and antenatal care visits. The number of antenatal care visits was divided into three categories: <4 antenatal care, 4–7 antenatal care visits, and ≥8 antenatal care.

### Statistical analysis

We generated descriptive analysis by frequency and percentage and stratified the sample by child age, place of residence, child gender, household wealth and maternal education to estimate the status of children with PEB. We included the sampling weights provided by the NFHS to ensure that the statistics were representative of the Indian households. Besides descriptive analysis, we conducted a series of multivariable logistic regressions to estimate the odds ratios (ORs) of the explanatory variables on PEB. We performed a sensitivity analysis in children receiving PEB and water in order to demonstrate the robustness of our findings. Before the multivariate analysis, we used variance inflation factors (VIF) to test for multicollinearity. We generated regression for each single wave of NFHS, in which we adjusted for breastfeeding initiation, drinking water source, sanitation facility, and antenatal care visits (mutually adjusted model). Besides, we developed sensitivity analyses based on adjusted variables in multivariate logistic regression plus child age and gender, respectively (single adjusted models). We used Stata software, version 17.0 (Stata Corporation LLC, College Station, USA) for all analysis procedures. All statistical tests were two-sided and *P* < 0.05 was considered to determine statistical significance.

## RESULTS

A total of 184 891 children aged 6–23 months were involved in this study, including 17 424 children from the survey of 1992–93, 14 188 from 1998–99, 14 422 from 2005–06, 74 132 from 2015–16, and 64 725 from 2019–21. In 1992–93, there were 4.0% (703) of the children who received PEB, which reduced to 2.4% (349) in 2005–06 and then increased to 7.7% (5002) in 2019–21. The rate of children who received PEB and water fluctuated rose from 11.1 (1929) to 14.2% (9219) from 1992–92 to 2019–21 (**Table 1**). Over time, the percentage of children from the poorest household wealth increased from 17.7 (3092) in 1992–93 to 26.2% (16 988) in 2019–21. Between 1992–93 and 2019–21, the percentage of mothers with no maternal education significantly decreased from 57.5 (10 022) to 19.3% (12 509), while the percentage of mothers with secondary education increased from 22.2 (3870) to 53.4% (34 586).

**Table 1:** Summary table of observation characteristics of children aged 6–23 months,1992–2021 (n = 184 891)

	1992–93 (n = 17 424)	1998–99 (n = 14 188)	2005–06 (n = 14 422)	2015–16 (n = 74 132)	2019–21 (n = 64 725)
Prolonged exclusive breastfeeding (PEB)					
*Yes*	703 (4.0%)	573 (4.0%)	349 (2.4%)	4536 (6.1%)	5002 (7.7%)
*No*	16 721 (96.0%)	13 615 (96.0%)	14 073 (97.6%)	69 596 (93.9%)	59 723 (92.3%)
PEB plus water					
*Yes*	1929 (11.1%)	1942 (13.7%)	1078 (7.5%)	10 143 (13.7%)	9219 (14.2%)
*No*	15 495 (88.9%)	12 246 (86.3%)	13 344 (92.5%)	63 989 (86.3%)	55 506 (85.8%)
Child’s age, months					
*6–8*	3281 (18.8%)	2589 (18.2%)	2602 (18.0%)	13 073 (17.6%)	10 831 (16.7%)
*9–11*	2543 (14.6%)	2121 (14.9%)	2294 (15.9%)	12 131 (16.4%)	10 713 (16.6%)
*12–23*	11 600 (66.6%)	9478 (66.8%)	9526 (66.1%)	48 928 (66.0%)	43 181 (66.7%)
Child gender					
*Male*	8915 (51.2%)	7421 (52.3%)	7543 (52.3%)	38 749 (52.3%)	33 469 (51.7%)
*Female*	8509 (48.8%)	6767 (47.7%)	6879 (47.7%)	35 383 (47.7%)	31 256 (48.3%)
Type of residence					
*Urban*	4829 (27.7%)	3843 (27.1%)	5478 (38.0%)	17 584 (23.7%)	13 086 (20.2%)
*Rural*	12 595 (72.3%)	10 345 (72.9%)	8944 (62.0%)	56 548 (76.3%)	51 639 (79.8%)
Household wealth quintile					
*1, poorest*	3092 (17.7%)	2547 (18.0%)	2433 (16.9%)	18 833 (25.4%)	16 988 (26.2%)
*2*	3157 (18.1%)	2605 (18.4%)	2604 (18.1%)	17 213 (23.2%)	14 969 (23.1%)
*3*	3452 (19.8%)	2998 (21.1%)	2896 (20.1%)	15 072 (20.3%)	12 792 (19.8%)
*4*	4183 (24.0%)	3273 (23.1%)	3271 (22.7%)	12 648 (17.1%)	11 067 (17.1%)
*5, richest*	3540 (20.3%)	2765 (19.5%)	3218 (22.3%)	10 366 (14.0%)	8909 (13.8%)
Maternal education					
*No schooling*	10 022 (57.5%)	6822 (48.1%)	5416 (37.6%)	21 012 (28.3%)	12 509 (19.3%)
*Primary*	2791 (16.0%)	2291 (16.1%)	1993 (13.8%)	10 360 (14.0%)	7621 (11.8%)
*Secondary*	3870 (22.2%)	3703 (26.1%)	5739 (39.8%)	34 924 (47.1%)	34 586 (53.4%)
*College or higher*	686 (3.9%)	1367 (9.6%)	1273 (8.8%)	7836 (10.6%)	10 009 (15.5%)
*Missing*	55 (0.3%)	5 (0.0%)	1 (0.0%)	0 (0.0%)	0 (0.0%)
Breastfeeding initiation					
*≥1 h of birth*	14 763 (84.7%)	10 970 (77.3%)	9391 (65.1%)	40 178 (54.2%)	34 921 (54.0%)
*<1 h of birth*	1993 (11.4%)	2702 (19.0%)	4453 (30.9%)	31 008 (41.8%)	27 058 (41.8%)
*Missing*	668 (3.8%)	516 (3.6%)	578 (4.0%)	2946 (4.0%)	2746 (4.2%)
Drinking water source					
*Unsafe*	683 (3.9%)	566 (4.0%)	2197 (15.2%)	6749 (9.1%)	4247 (6.6%)
*Safe*	16 718 (95.9%)	13 622 (96.0%)	11 201 (77.7%)	63 092 (85.1%)	56 019 (86.5%)
*Missing*	23 (0.1%)	0 (0.0%)	1024 (7.1%)	4291 (5.8%)	4459 (6.9%)
Sanitation facility					
*Not improved*	11 908 (68.3%)	8717 (61.4%)	7121 (49.4%)	34 446 (46.5%)	15 722 (24.3%)
*Improved*	5504 (31.6%)	5471 (38.6%)	6265 (43.4%)	35 395 (47.7%)	45 766 (70.7%)
*Missing*	12 (0.1%)	0 (0.0%)	1036 (7.2%)	4291 (5.8%)	3237 (5.0%)
Antenatal care visits					
*<4*	4830 (27.7%)	4616 (32.5%)	4667 (32.4%)	23 068 (31.1%)	18 722 (28.9%)
*4–7*	3552 (20.4%)	3436 (24.2%)	3937 (27.3%)	22 753 (30.7%)	24 382 (37.7%)
*≥8*	1464 (8.4%)	1342 (9.5%)	2174 (15.1%)	9421 (12.7%)	8949 (13.8%)
*Missing*	7578 (43.5%)	4794 (33.8%)	3644 (25.3%)	18 890 (25.5%)	12672 (19.6%)

We examined the allocation of children with/without PEB in 2019–21 (**Table 2**). Of the 64 725 children included in the analysis, there were 5002 children experiencing PEB. Among the exclusively breastfed children, there were 46.3% aged 6–8 months, 19.9% aged 9–11 months and 33.8% aged 12–23 months, respectively. Generally speaking, children who were experiencing PEB were more likely to concentrate in children younger than 12 months, among poorer households, in rural areas, and with secondary educated mothers. To illustrate, of children who were receiving PEB, 83.1% of them were living in rural areas, 30.4% of them were concentrated among the poorest households, and children from the richest households represented only 10.4%; moreover, the maternal education distribution in PEB group was 23.9% illiterate, 11.8% primary education, 51.8% secondary education, 12.5% college or above education.

**Table 2:** The allocation of the sample by the status of prolonged exclusive breastfeeding, 2019–21 (n = 64 725)*

	Prolonged exclusive breastfeeding		
	**Yes**	**No**	***P*-value**
n	5002	59 723	
Child’s age, months			<0.001
*6–8*	2316 (46.3%)	8515 (14.3%)	
*9–11*	994 (19.9%)	9719 (16.3%)	
*12–23*	1692 (33.8%)	41 489 (69.5%)	
Child gender			0.830
*Male*	2594 (51.9%)	30 875 (51.7%)	
*Female*	2408 (48.1%)	28 848 (48.3%)	
Type of residence			<0.001
*Urban*	847 (16.9%)	12 239 (20.5%)	
*Rural*	4155 (83.1%)	47 484 (79.5%)	
Household wealth quintile			<0.001
*1, poorest*	1519 (30.4%)	15 469 (25.9%)	
*2*	1272 (25.4%)	13 697 (22.9%)	
*3*	953 (19.1%)	11 839 (19.8%)	
*4*	737 (14.7%)	10 330 (17.3%)	
*5, richest*	521 (10.4%)	8388 (14.0%)	
Maternal education			<0.001
*No schooling*	1193 (23.9%)	11 316 (18.9%)	
*Primary*	592 (11.8%)	7029 (11.8%)	
*Secondary*	2593 (51.8%)	31 993 (53.6%)	
*College or higher*	624 (12.5%)	9385 (15.7%)	
Breastfeeding initiation			<0.001
*≥1 h of birth*	3170 (63.4%)	31 751 (53.2%)	
*<1 h of birth*	1814 (36.3%)	25 244 (42.3%)	
*Missing*	18 (0.4%)	2728 (4.6%)	
Drinking water source			0.460
*Unsafe*	342 (6.8%)	3905 (6.5%)	
*Safe*	4334 (86.6%)	51 685 (86.5%)	
*Missing*	326 (6.5%)	4133 (6.9%)	
Sanitation facility			<0.001
*Not improved*	1374 (27.5%)	14 348 (24.0%)	
*Improved*	3389 (67.8%)	42 377 (71.0%)	
*Missing*	239 (4.8%)	2998 (5.0%)	
Antenatal care visits			<0.001
*<4*	1652 (33.0%)	17 070 (28.6%)	
*4–7*	1875 (37.5%)	22 507 (37.7%)	
*≥8*	453 (9.1%)	8496 (14.2%)	
*Missing*	1022 (20.4%)	11 650 (19.5%)	

*Values are reported as n (%).

**Figure 1:**
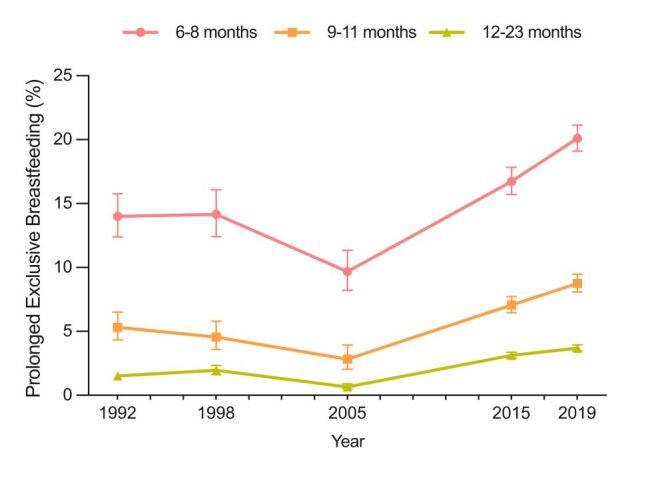
Percentage of children with prolonged exclusive breastfeeding between 1992–93 and 2019–21 by age groups.

The trends in the rate of PEB among children by age group are presented in **Figure 1**. We found that the proportion of PEB by child age showed a similar fluctuating upward trend as the overall trend. Specifically, there were 14.0% (95% CI = 12.4–15.8) of children aged 6–8 months old exclusively breastfed in 1992–93, which reduced to 9.7% (95% CI = 8.2–11.4) in 2005–06, and afterward, increased to 20.1% (95% CI = 19.1–21.1) in 2019-21. Similar trends were also observed for children aged 9–11 months and 12–23 months old.

**Figure 2:**
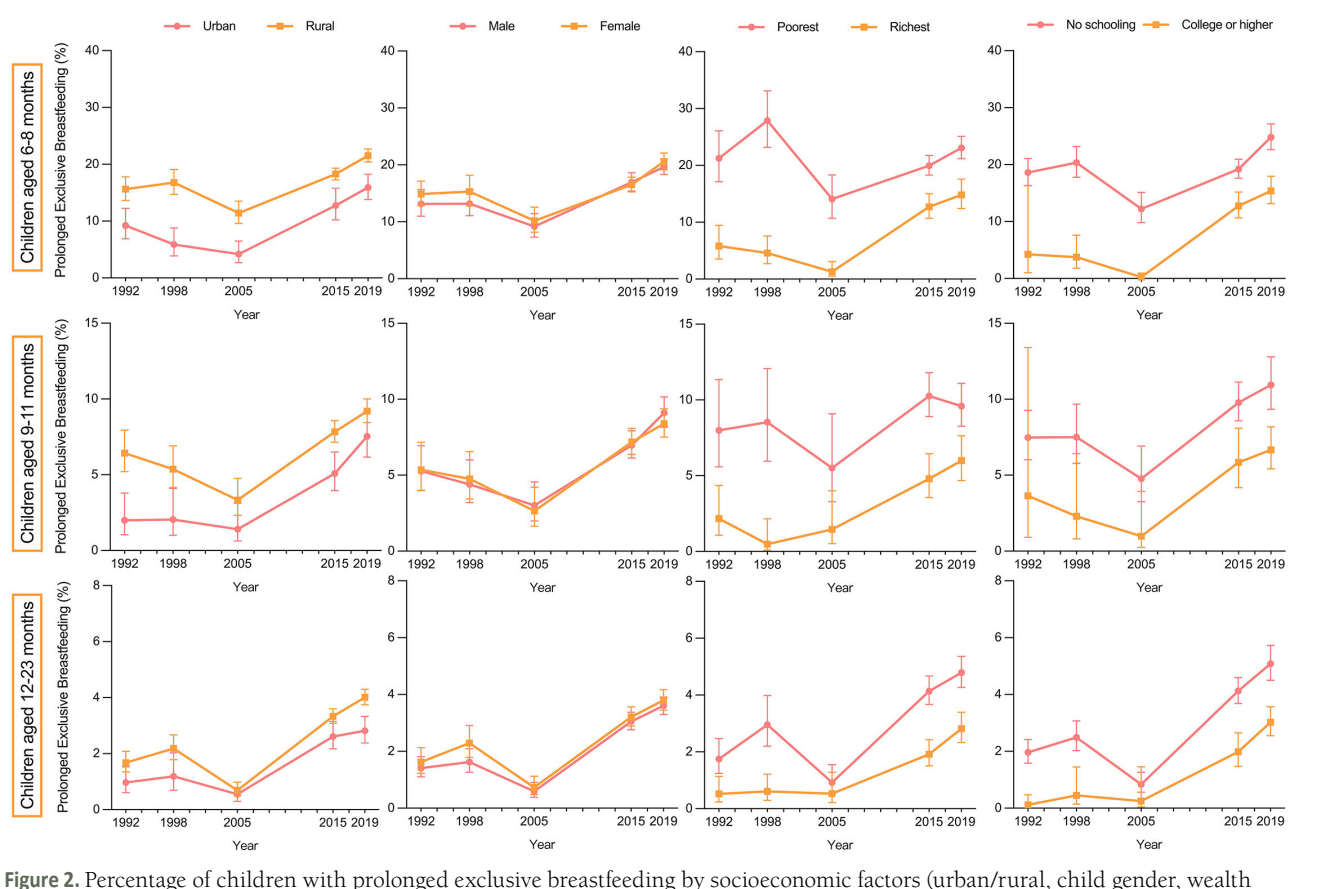
Percentage of children with prolonged exclusive breastfeeding by socioeconomic factors (urban/rural, child gender, wealth quintile, maternal education level) between 1992–93 and 2019–21.

**Figure 2** shows the tendency of the percentage of PEB by socioeconomic factors in different age groups. In all age groups, children living in rural areas, from poorer households, or with lower-educated mothers were more likely to be exclusively breastfed in all years. For instance, there were 23.09% (95% CI = 21.18–25.12) of the poorest children exclusively breastfed in 2019–21, compared to 14.81% (95% CI = 12.41–17.58) of the richest in children aged six-eight months. Consistent with the overall trend, we found that for subgroups by socioeconomic factors, the proportion of children receiving PEB fluctuated upward over time. The increase was particularly notable among children with better-educated mothers. Among children aged 6–8 months whose mothers were with college or above education, merely 4.25% (95% CI = 1.00–16.27) of them received PEB in 1992-93; the percentage largely increased to 15.39% (95% CI = 13.14–17.94) in 2019–21.

**Table 3** indicates the associations between breastfeeding practices and socioeconomic factors between 1992–2021 from mutually adjusted model. There is none showing evidence for multicollinearity (all VIF values <10). We found that compared to children older than 12 months, younger infants are more likely to be exclusively breastfed, and the younger they are, the higher the odds of PEB. To specify, infants aged six-eight months were 6.39 times more likely to experience PEB than infants aged 12–23 months (95% CI = 5.57–7.34) in 2019–21. Over the same period, infants aged 9–11 months were 2.28 times more likely to experience PEB than infants aged 12–23 months (95% CI = 1.97–2.64). In general, children from poorer households, and children with less educated mothers were independent predictors of children receiving PEB. The association of household wealth was particularly strong. In particular, compared to the richest children, the poorer ones had significantly higher odds to be exclusively breastfed with an OR of 1.33 after covariate adjustment (95% CI = 1.09–1.61). Maternal education also showed a strong association with PEB. In detail, compared to the children with higher education mothers, the ones whose mothers had no education were 21% more likely to receive PEB after adjusting covariates (OR = 1.21; 95% CI = 1.01–1.44). In addition, wealth and maternal education were not associated with PEB prior to 2005. However, after that, children from poorer families are more vulnerable to being prolonged exclusively breastfed, while children of illiterate mothers are more likely to experience PEB. When we additionally adjusted for children’s age and gender separately, the results remained stable (Table S1 in the **Online Supplementary Document**). Children who exclusively consumed breastmilk and water had a similar result as those who experienced PEB (Tables S2–S3 in the **Online Supplementary Document**).

**Table 3:** Association between prolonged exclusive breastfeeding and socioeconomic factors between 1992–93 and 2019–21 from mutually adjusted model*

	Prolonged exclusive breastfeeding, OR (95% CI)					
	**1992–93**	**1998–99**	**2005–06**	**2015–16**	**2019–21**	***P* for interaction**
Child’s age, months						<0.001
*6–8*	9.70 (6.62–14.22)†	12.51 (8.39–18.66)†	24.66 (14.11–43.10)†	6.39 (5.57–7.34)†	6.69 (6.03–7.44)†	
*9–11*	3.15 (1.98–5.03)†	2.93 (1.78–4.81)†	6.21 (3.12–12.34)†	2.28 (1.97–2.64)†	2.38 (2.10–2.70)†	
*12–23*	Ref.	Ref.	Ref.	Ref.	Ref.	
Child gender						0.936
*Male*	Ref.	Ref.	Ref.	Ref.	Ref.	
*Female*	1.09 (0.81–1.47)	1.15 (0.84–1.56)	0.96 (0.67–1.38)	1.04 (0.92–1.17)	1.02 (0.93–1.13)	
Type of residence						0.067
*Urban*	Ref.	Ref.	Ref.	Ref.	Ref.	
*Rural*	0.93 (0.63–1.38)	1.06 (0.59–1.89)	1.33 (0.78–2.28)	1.07 (0.87–1.32)	1.10 (0.95–1.28)	
Household wealth quintile						<0.001
*1, poorest*	1.51 (0.75–3.06)	2.06 (0.92–4.59)	3.12 (1.22–8.01)‡	1.37 (1.04–1.80)‡	1.23 (1.00–1.51)‡	
*2*	1.48 (0.80–2.75)	1.39 (0.63–3.02)	2.04 (0.82–5.04)	1.37 (1.09–1.73)§	1.33 (1.09–1.61)§	
*3*	1.44 (0.76–2.71)	1.06 (0.51–2.18)	1.32 (0.56–3.11)	1.29 (1.02–1.62)‡	1.20 (0.99–1.45)	
*4*	1.02 (0.58–1.79)	0.97 (0.48–1.93)	0.98 (0.44–2.15)	1.13 (0.90–1.40)	1.10 (0.91–1.32)	
*5, richest*	Ref.	Ref.	Ref.	Ref.	Ref.	
Maternal education						<0.001
*No schooling*	1.78 (0.50–6.29)	2.05 (0.91–4.60)	5.39 (1.55–18.70)§	1.07 (0.86–1.33)	1.21 (1.01–1.44)‡	
*Primary*	0.78 (0.21–2.87)	1.41 (0.63–3.17)	4.01 (1.11–14.51)‡	1.08 (0.81–1.44)	1.19 (0.97–1.46)	
*Secondary*	0.60 (0.16–2.26)	0.99 (0.45–2.19)	3.47 (1.08–11.16)‡	1.03 (0.85–1.25)	1.03 (0.88–1.20)	
*College or higher*	Ref.	Ref.	Ref.	Ref.	Ref.	

CI – confidence interval, OR – odds ratio, Ref. – reference

*Adjusted for breastfeeding initiation, drinking water source, sanitation facility, and antenatal care visits.

†*P* < 0.001.

‡*P* < 0.05.

§*P* < 0.01.

## DISCUSSION

Using five waves of nationally representative surveys conducted between 1992–93 and 2019–21, our study generated three salient findings: first, we found that PEB was prevalent among children aged 6–23 months, with 20.10% of the children aged six-eight months old were exclusively breastfed; even among children aged 12 months or older, 3.69% were exclusively breastfed in 2019–21. Second, over the past two decades, the prevalence of prolonged exclusive breastfeeding in India increased gradually for all age groups. Last, the disadvantaged children (living in rural, the poorer, with less educated mothers) were more likely to suffer from PEB basically in all studied years.

In this study, we found PEB to be very prevalent in India. Although the community has been emphasising the importance of timely complementary feeding, what is less known is that PEB is not only prevalent within six-eight months but also occurs in children beyond 12 months of age. In recent years, the significance of exclusive breastfeeding has been gradually recognised by the general public and has been extensively promoted [31,32]. However, policymakers need to be vigilant against promotional biases and emphasise the importance of the time frame, namely, exclusive breastfeeding within the first six months of life. To avoid the occurrence of extremely exclusive breastfeeding, it is also crucial to promote the gradual introduction of complementary foods after six months of age. The timely introduction of complementary foods is very important for children aged 6–8 months, which is a critical window for the introduction of solid foods. Starting at six months, infant's oral muscles begin to develop, and using different textures of food at this time can exercise their chewing ability and facilitate future language development [33]. In addition, the late introduction of complementary foods was associated with a lot of nutritional inadequacy problems like iron, zinc, and vitamin D deficiency [34-36]. This suggests that we need to promote breastfeeding while also increasing education on the timely introduction of complementary foods.

This study found that the proportion of children aged 6–23 months old who were exclusively breastfed decreased from 1998–99 to 2005–06, followed by a substantial increase in subsequent years. We suspect that it may be related to the heightened emphasis on the promotion of exclusive breastfeeding through widespread advertisement with less focus on proper feeding practices. Historically, in the late 90s and early 2000s, India enacted a compendium of policy initiatives aimed at bolstering breastfeeding practices, including the National Guidelines on Infant and Young Child Feeding, the National Plan of Action on Nutrition, and the Infant Milk Substitutes, Feeding Bottles and Infant foods [37]. Globally, in January 1998, during its 101st session, the Executive Board of the WHO championed a reinvigorated commitment to the nutritional well-being of infants and young children, underscoring the significance of breastfeeding and complementary feeding. Subsequently, in 2003, the WHO, in collaboration with the United Nations Children's Fund (UNICEF), devised the Global Strategy for Infant and Young Child Feeding. This strategy aimed to emphasise enhancing the nutrition, growth, development, and health of infants and young children through optimal feeding practices, ultimately aiming to bolster their overall survival. These legislative efforts were supplemented by a multitude of campaigns and outreach programmes, all endeavoring to protect, advocate for, and fortify the practice of exclusive breastfeeding during the first six months of life. The powerful advertisement of the benefits of exclusive breastfeeding has increased the rate of exclusive breastfeeding for children under six months, but it may also hinder the awareness of the importance of other food sources, as well as when and how other foods should be introduced to the children [38].

Similar to the previous studies [19,39], we found that children from more advantaged households were less likely to suffer from poor breastfeeding practices like PEB. Previous studies showed that maternal education could promote proper feeding practices, including proper timing for initiation of complementary feeding, continuation of breastfeeding after introduction of semisolid foods, hygiene, composition, amount, consistency, and frequency of feeding, and feeding of the infant during or after illness; maternal education also showed notable effects on nutrition intake, and dietary diversity [40–45]. Education interventions targeting primary caregivers also showed an effect on children’s weight, length, mid-upper-arm circumference, stunting, wasting, and underweight, which might potentially result from better nutrition intake [41,42,45,46].

The study has some data-related limitations. First, NFHS-1 did not include a detailed list of what food has been given to the children, but only a rather broad question on whether ‘gave child solid or mushy food’. The mothers might be subject to forgetting an item given to the child without being given a list of food. Second, although 24-hour maternal recall is the method recommended by WHO to assess child dietary intake [5] and has been widely used in programme monitoring and evaluations [19,39,47], the measurement completely relies on self-report and could be prone to maternal recall bias or social desirability in reporting. Third, the NFHS data did not use weights to correct survival selection bias. Children who were not alive when the survey was taken were excluded from the questions on breastfeeding, and treated as not currently breastfeeding in the numerator and included in the denominator. Such children may be more likely to be from the disadvantaged population and suffer from the poorest dietary intake. However, given the data we have, we cannot fully address this issue. Fourth, the data set does not have sufficient information for us to assess the changes in frequency and quality of food consumption, not does it allow us to include information on nutrition knowledge or exposure to educational interventions. Finally, with the repeated cross-sectional data provided by the NFHS, we were unable to identify any causal effect between socioeconomic status or other covariates and feeding practices.

## CONCLUSIONS

This is the first study exploring the changes in PEB practices in India. Our results indicated that, in order to meet children’s developmental requirements, India is in urgent need to promote healthy feeding practices and reduce food deprivation in children over six months. Educational interventions targeting the mothers might effectively be better explored to help the caregivers make better and healthier feeding choices for the children.

## Additional material


Online Supplementary Document
