# Supplementary material for: Prevalence, trend, and inequality of prolonged exclusive breastfeeding among children aged 6–23 months old in India from 1992–2021: A cross-sectional study of nationally representative, individual-level data
Source: J Glob Health. 2024 Feb 9;14:04026. doi: 10.7189/jogh.14.04026 (PMC10854209; doi:10.7189/jogh.14.04026)
Supplement: Online Supplementary Document [file jogh-14-04026-s001.pdf]

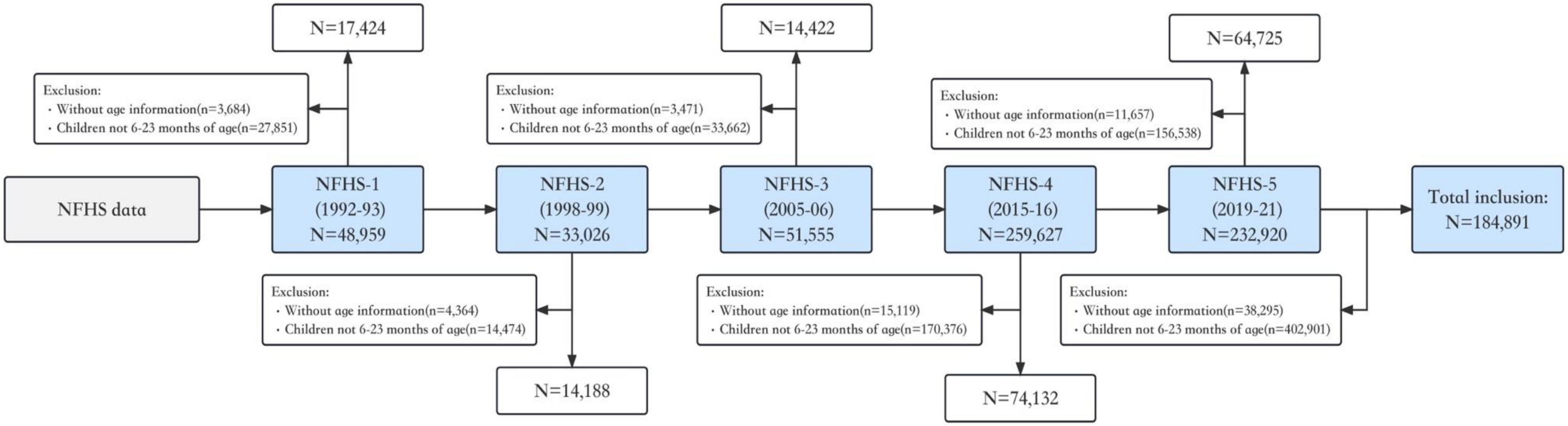

Figure S1. Flowchart of participants throughout the study

Table S1. Association between prolonged exclusive breastfeeding and socioeconomic factors between 1992-93 and 2019-21 from single adjusted model

	Prolonged exclusive breastfeeding, OR (95% CI)					
	**1992-93**	**1998-99**	**2005-06**	**2015-16**	**2019-21**	***P* for interaction**
**Model 1†**						
**Type of residence**						
Urban	Ref.	Ref.	Ref.	Ref.	Ref.	0.067
Rural	0.93(0.63,1.38)	1.06(0.60,1.90)	1.34(0.78,2.28)	1.07(0.87,1.32)	1.10(0.95,1.28)	
**Household wealth quintile**						
1, poorest	1.53(0.75,3.10)	2.07(0.93,4.59)	3.12(1.22,8.00)* §	1.37(1.04,1.80)*	1.22(0.99,1.51)	<0.001
2	1.50(0.80,2.78)	1.38(0.63,3.00)	2.03(0.82,5.02)	1.37(1.09,1.73)**	1.33(1.09,1.61)**	
3	1.45(0.77,2.73)	1.06(0.52,2.19)	1.31(0.56,3.11)	1.29(1.02,1.62)*	1.20(0.99,1.45)	
4	1.02(0.58,1.80)	0.96(0.48,1.91)	0.97(0.44,2.14)	1.13(0.91,1.40)	1.10(0.91,1.32)	
5, richest	Ref.	Ref.	Ref.	Ref.	Ref.	
**Maternal education**						
No schooling	1.78(0.50,6.29)	2.05(0.91,4.62)	5.38(1.55,18.71)**	1.07(0.86,1.33)	1.21(1.01,1.44)*	<0.001
Primary	0.78(0.21,2.89)	1.41(0.63,3.18)	4.01(1.11,14.51)*	1.08(0.81,1.44)	1.19(0.97,1.46)	
Secondary	0.60(0.16,2.25)	1.00(0.45,2.19)	3.46(1.08,11.15)*	1.03(0.84,1.25)	1.03(0.88,1.20)	
College or higher	Ref.	Ref.	Ref.	Ref.	Ref.	
**Model 2‡**						
**Type of residence**						
Urban	Ref.	Ref.	Ref.	Ref.	Ref.	0.0908
Rural	0.90(0.62,1.33)	1.07(0.59,1.93)	1.31(0.76,2.25)	1.09(0.88,1.35)	1.14(0.99,1.32)	
**Household wealth quintile**						<0.001
1, poorest	1.35(0.69,2.66)	1.83(0.79,4.24)	3.06(1.23,7.63)*	1.31(1.00,1.71)*	1.19(0.97,1.45)	

2	1.44(0.79,2.62)	1.31(0.58,2.93)	1.89(0.79,4.54)	1.35(1.08,1.70)**	1.29(1.07,1.55)**	
3	1.32(0.70,2.47)	1.02(0.48,2.16)	1.32(0.57,3.09)	1.25(0.99,1.58)	1.16(0.97,1.39)	
4	0.99(0.57,1.71)	1.02(0.51,2.05)	1.05(0.48,2.31)	1.09(0.88,1.35)	1.06(0.89,1.27)	
5, richest	Ref.	Ref.	Ref.	Ref.	Ref.	
**Maternal education**						
No schooling	1.77(0.50,6.29)	2.09(0.93,4.71)	4.64(1.37,15.68)*	1.05(0.84,1.30)	1.18(1.00,1.38)*	
Primary	0.76(0.20,2.83)	1.43(0.63,3.22)	4.02(1.15,13.99)*	1.04(0.77,1.39)	1.16(0.95,1.41)	<0.001
Secondary	0.58(0.15,2.21)	1.02(0.47,2.22)	3.33(1.05,10.52)*	1.01(0.83,1.23)	1.01(0.87,1.17)	
College or higher	Ref.	Ref.	Ref.	Ref.	Ref.	

Abbreviations: OR – odds ratio.

† Adjusted for children age, breastfeeding initiation, drinking water source, sanitation facility, antenatal care visits.

‡ Adjusted for children gender, breastfeeding initiation, drinking water source, sanitation facility, antenatal care visits.

§* $P<0.05$, ** $P<0.01$, *** $P<0.001$.

Table S2. Association between children receiving breastmilk plus water and socioeconomic factors between 1992-93 and 2019-21 from mutually adjusted model†

	Breastfeeding plus water, OR (95% CI)					
	**1992-93**	**1998-99**	**2005-06**	**2015-16**	**2019-21**	***P* for interaction**
**Child's age, mo**						
6-8	10.27(8.17,12.91)***[b]	10.57(8.63,12.94)***	16.05(12.32,20.92)***	8.56(7.79,9.40)***	6.54(6.02,7.12)***	<0.001
9-11	3.30(2.50,4.36)***	3.91(3.04,5.03)***	5.81(4.20,8.05)***	3.24(2.93,3.59)***	2.48(2.26,2.72)***	
12-23	Ref.	Ref.	Ref.	Ref.	Ref.	
**Child gender**						
Male	Ref.	Ref.	Ref.	Ref.	Ref.	0.562
Female	1.11(0.92,1.34)	1.00(0.84,1.20)	1.15(0.94,1.42)	1.05(0.97,1.13)	1.00(0.93,1.07)	
**Type of residence**						
Urban	Ref.	Ref.	Ref.	Ref.	Ref.	0.680
Rural	0.93(0.71,1.22)	0.84(0.66,1.08)	0.86(0.64,1.14)	1.03(0.91,1.16)	1.09(0.97,1.21)	
**Household wealth quintile**						
1, poorest	1.23(0.77,1.95)	1.89(1.22,2.93)**	2.46(1.41,4.30)**	1.44(1.20,1.74)***	1.38(1.17,1.63)***	
2	1.26(0.83,1.91)	1.19(0.78,1.81)	1.45(0.85,2.46)	1.30(1.10,1.53)**	1.39(1.20,1.62)***	<0.001
3	1.13(0.76,1.68)	1.29(0.88,1.89)	1.77(1.08,2.91)*	1.23(1.04,1.44)*	1.25(1.08,1.44)**	
4	1.09(0.76,1.56)	0.97(0.70,1.35)	1.25(0.81,1.93)	1.16(0.99,1.35)	1.13(0.98,1.31)	
5, richest	Ref.	Ref.	Ref.	Ref.	Ref.	
**Maternal education**						
No schooling	3.67(1.32,10.16)*	2.74(1.75,4.29)***	2.01(1.07,3.76)*	1.55(1.32,1.82)***	1.45(1.25,1.67)***	<0.001
Primary	1.97(0.72,5.40)	1.73(1.10,2.71)*	1.76(0.92,3.37)	1.28(1.06,1.54)**	1.22(1.04,1.43)*	
Secondary	1.58(0.57,4.37)	1.65(1.09,2.50)*	1.05(0.59,1.86)	1.25(1.08,1.44)**	1.08(0.96,1.22)	

College or higher	Ref.	Ref.	Ref.	Ref.	Ref.

Abbreviations: OR – odds ratio.

† Adjusted for breastfeeding initiation, drinking water source, sanitation facility, and antenatal care visits.

‡* *P*<0.05, ** *P* <0.01, *** *P* <0.001.

Table S3. Association between children receiving breastmilk plus water and socioeconomic factors between 1992-93 and 2019-21 from single adjusted model

	Breastfeeding plus water, OR (95% CI)					
	**1992-93**	**1998-99**	**2005-06**	**2015-16**	**2019-21**	***P* for interaction**
**Model 1†**						
**Type of residence**						
Urban	Ref.	Ref.	Ref.	Ref.	Ref.	0.680
Rural	0.93(0.71,1.22)	0.84(0.66,1.08)	0.85(0.64,1.13)	1.03(0.91,1.16)	1.09(0.97,1.21)	
**Household wealth quintile**						
1, poorest	1.24(0.78,1.97)	1.89(1.22,2.93)** §	2.47(1.41,4.32)**	1.45(1.20,1.74)***	1.38(1.17,1.63)***	<0.001
2	1.27(0.83,1.93)	1.19(0.78,1.81)	1.46(0.86,2.48)	1.30(1.10,1.54)**	1.39(1.20,1.62)***	
3	1.14(0.77,1.69)	1.29(0.88,1.89)	1.78(1.08,2.92)*	1.23(1.04,1.44)*	1.25(1.08,1.44)**	
4	1.10(0.77,1.57)	0.97(0.70,1.35)	1.26(0.81,1.95)	1.16(0.99,1.35)	1.13(0.98,1.31)	
5, richest	Ref.	Ref.	Ref.	Ref.	Ref.	
**Maternal education**						
No schooling	3.67(1.33,10.15)*	2.74(1.75,4.29)***	2.01(1.08,3.76)*	1.55(1.32,1.82)***	1.45(1.25,1.67)***	<0.001
Primary	1.98(0.72,5.41)	1.73(1.10,2.71)*	1.77(0.93,3.38)	1.28(1.06,1.54)**	1.22(1.04,1.43)*	
Secondary	1.58(0.57,4.35)	1.65(1.09,2.50)*	1.06(0.60,1.86)	1.24(1.08,1.44)**	1.08(0.96,1.22)	
College or higher	Ref.	Ref.	Ref.	Ref.	Ref.	
**Model 2‡**						
**Type of residence**						
Urban	Ref.	Ref.	Ref.	Ref.	Ref.	0.751
Rural	0.91(0.71,1.16)	0.87(0.68,1.13)	0.87(0.66,1.15)	1.05(0.94,1.19)	1.12(1.01,1.24)*	
**Household wealth quintile**						
1, poorest	1.07(0.69,1.66)	1.66(1.07,2.55)*	2.32(1.38,3.91)**	1.35(1.13,1.61)**	1.33(1.14,1.54)***	0.002
2	1.18(0.80,1.75)	1.16(0.76,1.76)	1.39(0.85,2.29)	1.27(1.08,1.49)**	1.34(1.16,1.54)***	

3	1.02(0.70,1.48)	1.25(0.85,1.84)	1.71(1.07,2.72)*	1.19(1.01,1.39)*	1.20(1.05,1.38)**	
4	1.04(0.74,1.46)	1.04(0.76,1.43)	1.37(0.91,2.07)	1.11(0.96,1.29)	1.09(0.95,1.25)	
5, richest	Ref.	Ref.	Ref.	Ref.	Ref.	
**Maternal education**						
No schooling	3.41(1.24,9.37)*	2.59(1.67,4.03)***	1.62(0.90,2.93)	1.45(1.24,1.69)***	1.36(1.19,1.55)***	
Primary	1.88(0.69,5.12)	1.74(1.11,2.73)*	1.56(0.85,2.88)	1.19(0.99,1.43)	1.18(1.01,1.37)*	<0.001
Secondary	1.50(0.54,4.11)	1.65(1.10,2.49)*	1.00(0.58,1.73)	1.20(1.04,1.38)*	1.06(0.94,1.18)	
College or higher	Ref.	Ref.	Ref.	Ref.	Ref.	

Abbreviations: OR – odds ratio.

† Adjusted for children age, breastfeeding initiation, drinking water source, sanitation facility, antenatal care visits.

‡ Adjusted for children gender, breastfeeding initiation, drinking water source, sanitation facility, antenatal care visits.

§* *P*<0.05, ** *P* <0.01, *** *P* <0.001.
